# Dietary Zinc Deficiency in Rodents: Effects on T-Cell Development, Maturation and Phenotypes

**DOI:** 10.3390/nu4060449

**Published:** 2012-06-06

**Authors:** Heather J. Blewett, Carla G. Taylor

**Affiliations:** 1 Canadian Centre for Agri-Food Research in Health and Medicine, Winnipeg, MB R2H 2A6, Canada; Email: hblewett@sbrc.ca; 2 Department of Human Nutritional Sciences, University of Manitoba, Winnipeg, MB R3T 2N2, Canada

**Keywords:** zinc, immune function, T-cell development, T-cell maturation, recent thymic emigrants, p56^lck^, corticosterone, flow cytometry, rats, mice

## Abstract

Zinc deficiency is one of the leading risk factors for developing disease and yet we do not have a clear understanding of the mechanisms behind the increased susceptibility to infection. This review will examine the interrelationships among the hypothalamus-pituitary-adrenal stress axis, p56^lck^, and T-cell maturation in both zinc deficiency and responses during zinc repletion. We will highlight differences between the adult mouse model (wasting malnutrition) and growing rat model (stunting malnutrition) of dietary zinc deficiency and discuss the use of various controls to separate out the effects of zinc deficiency from the associated malnutrition. Elevated serum corticosterone in both zinc deficient and pair-fed rats does not support the hypothesis that zinc deficiency *per se* leads to corticosterone-induced apoptosis and lymphopenia. In fact, the zinc deficient rat does not have lymphopenia. Thymocytes from zinc deficient mice and rats have elevated levels of p56^lck^, a signalling protein with a zinc clasp structure, but this does not appear to affect thymocyte maturation. However, post-thymic T-cell maturation appears to be altered based on the lower proportion of splenic late thymic emigrants in zinc deficient rats. Fewer new T-cells in the periphery could adversely affect the T-cell repertoire and contribute to immunodeficiency in zinc deficiency.

## 1. Introduction

Nutrition plays an important role in the functioning of the immune system [1]. All cells in the body require certain nutrients to function properly, and the highly proliferative cells involved in immune defense are particularly vulnerable to nutrient deficiencies leaving them functionally compromised [2]. This review explores the essentiality of zinc in the development and proper functioning of T-cells which are involved in cell-mediated immune responses. 

## 2. Overview of Dietary Zinc

### 2.1. Functional Roles of Zinc

Zinc is known to play a role structurally or functionally in over 300 enzymes in the body and it is also involved in two other classes of proteins: metallothioneins and gene regulatory proteins [3]. In the cell, zinc containing proteins play a role in many functions ranging from metabolic pathways to regulation of gene expression [4]. One important group of zinc-containing proteins is the zinc-finger proteins. Zinc finger proteins are aptly named because zinc stabilizes small protein loops that resemble fingers, and this conformation allows the zinc finger to bind to DNA or other proteins [5]. Zinc finger proteins are found throughout the cell and may play a part in countless protein to protein interactions including signal transduction molecules in T-cells [6]. Since zinc is part of so many proteins necessary for signal transduction and proliferation, it comes as no surprise that dietary zinc has an influence on the highly proliferative immune system.

### 2.2. Zinc Body Pools

Plasma contains approximately 15.3 µmol Zn/L bound to α-macroglobulin and albumin, which represents <1% of total body zinc [3]. During zinc deficiency, plasma zinc concentration can drop by 25–88% depending on the length and amount of zinc restriction [7]; however, plasma zinc concentrations cannot be reliably used to diagnose zinc deficiency because it is not sensitive (does not detect marginal zinc deficiency) and it is not specific (acute infections can also lower plasma zinc concentrations) [8]. The other 99% of total body zinc is intracellular [3]. Intracellular zinc is often bound to metallothionein which has a short half-life, leading some to believe that it acts as a zinc pool [8]. However, there is no concrete evidence that any one tissue stores zinc. Some have looked to bone as a potential zinc pool since bone holds 30% of total body zinc [9]. Evidence shows that during initial zinc deficiency, there is a small pool of zinc that can be removed from bone (10–20%), and after that no more zinc can be removed without losing bone mass [9]. To date, femur zinc concentration is the most reliable assessment to evaluate long term zinc deficiency in animal models.

### 2.3. Dietary Zinc Deficiency

Dietary zinc deficiency occurs when there is an inadequate dietary zinc intake or a decreased ability to absorb zinc from the diet [10]. Because zinc is present in a wide variety of foods (particularly protein rich foods) dietary zinc deficiency was not a concern until the first documented cases of dietary zinc deficiency were reported in the 1960s [11]. A group of teenaged Middle Eastern boys presented with dwarfism, delayed sexual maturation, rough dry skin and increased susceptibility to infections [12]. These boys were not related, and the only commonality between them was a cereal based diet that was both low in zinc and high in phytates. Phytates, found in whole grain cereals, pulses and nuts, inhibit the absorption of zinc thereby increasing the risk of dietary zinc deficiency [13].

Although dietary zinc deficiency was originally thought to be a rare occurrence, it has recently been estimated that 1 out of 5 people in the world might not be consuming or absorbing enough zinc from their diet [14]. Studies from around the world have shown that zinc supplementation reduces the rates of diarrhea, pneumonia, malaria and mortality in children [15,16,17,18] suggesting that a small increase in dietary zinc intake could make a difference in the health of millions of people in this world. Besides dietary intake, there are diseases that affect the absorption of zinc from the diet and thus contribute to the prevalence of zinc deficiency in diseases such as renal disease, Crohn’s disease, sickle cell anemia, and acrodermatitis enteropathica [19].

Zinc deficiency is listed as one of the top 10 risk factors for developing disease in developing countries and it is among the top 20 risk factors on a global scale [20]. Zinc deficiency is a public health concern because it alters immune function leaving the population susceptible to disease. The research designed to elucidate how zinc deficiency affects T-cell development and maturation in the growing rat model will be discussed in upcoming sections. In order to understand the role of zinc in the immune system, a general discussion of the immune system is offered in the following section.

## 3. Overview of the Immune System and Its Assessment

### 3.1. The Immune Response

Our bodies provide the perfect environment for cells to live and function, including pathogens. The evolution of the immune system has provided us with a complex mechanism to protect us from death by infection [21]. Various analogies can be drawn between the components of the immune system and various defenses for protection of one’s home or property. The first line of defense against foreign antigens is the skin and mucous membranes that work much like the walls, windows and doors of a house to keep strangers on the street from entering the home [22]. Often homes are armed with an alarm system that sounds whenever an intruder enters. The alarm system is similar to the second line of defense called the innate immune response, which will respond to all foreign antigens nonspecifically [23]. For example, with a cut in the skin, microorganisms begin to invade the body and the immune system initiates an inflammatory response which summons the arrival of immune cells to destroy the pathogen [23]. The immune cells that arrive recognize that the pathogen is foreign and can distinguish between different classes of pathogens, but they don’t need to know that pathogen specifically. A third line of defense, called the adaptive immune response, is based on immune cells being able to recognize one specific pathogen [21]. This is like the fire alarm in your house, which will only sound when a fire has started and summons the appropriate aid. 

The adaptive immune response is mediated by B- and T-cells (or B- and T-lymphocytes) [22]. B-cells mature in the bone marrow, while T-cells leave the bone marrow to mature in the thymus before entering secondary lymphoid organs like the spleen, lymph nodes and gut associated lymphoid tissue where they respond to their specific antigen when stimulated [24]. 

Activation of B-cells results in the production of antibodies which then travel throughout the body and bind to all molecules that are similar to the antigen that initiated the immune response [24]. Once the antibodies have bound to the antigens, this will either inactivate the antigen, bind the antigens together to facilitate phagocytosis by macrophages, or destroy the antigen by activating the complement cascade [21]. This response is called the humoral response [21]. 

T-cells attack antigens more directly and comprise the cell-mediated response [21]. T-cells have two main subsets: cytotoxic and helper [25]. Determination of cell surface markers or clusters of differentiation (CD) using monoclonal antibodies and flow cytometry can distinguish the subtype of lymphocyte. CD4 can be used to identify helper T-cells while CD8 reveals cytotoxic T-cells [24]. 

Some of the classic characteristics of dietary zinc deficiency are thymic atrophy and lymphopenia which has focused attention on T-cells [26] and the rest of this review will pertain directly to this subset of immune cells.

### 3.2. T-Cell Maturation

The thymus gland is a bi-lobed organ situated above the heart. Each lobe can be divided into lobules and each lobule can be divided into an outer area (or cortex) and an inner area (or medulla) [27]. The most immature T-cells are found in the cortex and do not express the T-cell receptor (TCR):CD3 complex, CD4 or CD8 [27]. As the cells travel towards the medulla, they begin to express TCRαβ, CD4, and CD8 [27]. Before thymocytes become fully mature and express only CD4 or CD8 along with TCRαβ, they go through positive and negative selection [28]. This selection process ensures that cells that react strongly to ‘self’ major histocompatibility complexes (MHC) are removed by apoptosis and those that don’t recognize self MHC at all die of neglect [25]. Homeostasis is maintained when there is control over the amount of cells being made and the amount of cells being destroyed [29]. If too many cells are killed, leukopenia and immunodeficiency occurs [29]. If not enough lymphocytes are destroyed, autoimmunity can occur [29]. A malfunctioning thymus can therefore prevent production or proper functioning mature T-cells.

Intrathymic labeling using fluorescein isothiocyanate (FITC) [30] and thymus transplantation [31] have allowed researchers to identify and characterize differentiation of T-cells once they have been released from the thymus into the periphery. In rats, CD90 first appears on cortical thymocytes and is found on newly released T-cells (recent thymic emigrants) for approximately 3 days after T-cells are released from the thymus and are circulating in the blood or accumulating in the secondary lymphoid organs [30]. The ability to identify newly released thymocytes with CD90 is unique to the rat, and therefore positions the rat as the best model to study post-thymic development. CD90 plays a role in T-cell activation [32], and in the diabetes-prone BioBreeding rat, a high proportion of recent thymic emigrants are apoptotic [33]. RT6 and CD45RC are expressed as CD90 disappears (3–11 days post-thymus) and 76 days after T-cells have been released from the thymus they express either CD45RC or RT6 [31]. CD45RC is involved in T- and B-cell receptor signal transduction, and is often used to discriminate naïve from memory T-cells [34,35]. RT6.1 is expressed solely on peripheral T-cells and it is hypothesized that RT6.1 expression prevents apoptosis of recent thymic emigrants [36]. Cell surface markers like these have been useful to determine how dietary zinc deficiency alters T-cell maturation. 

### 3.3. Applications of Flow Cytometry

The rapid measurement of the physical and/or chemical characteristics of individual cells while they pass, in single file, through a laser beam of light is called flow cytometry [37,38]. As the cells pass through the laser, they scatter light in two ways. Forward scatter is the light deflected from the surface of the cell (indicates the size of the cell), and side scatter is the light deflected off internal structures of the cell (indicates granularity of the cell) [37,38]. Flow cytometers also measure the fluorescence emitted by fluorescent dyes. Cells are labeled using monoclonal antibodies that are directly bound to fluorochromes, or indirectly when a fluorochrome is conjugated to a secondary antibody that recognizes the unconjugated antibody [37,38]. There are numerous fluorochromes available, each producing a distinct color, and which can be used in combination to determine many characteristics of the cell simultaneously [39]. Flow cytometry is often used to identify cells based on their cell surface markers (*i.e.*, CD4 *vs.* CD8) and report the proportion of these cells in a sample population. Absolute cell numbers can also be obtained by adding a specific number of counting beads to a specific volume of sample. The volume of sample can be determined based on the number of beads counted, allowing the number of cells per milliliter to be calculated. Flow cytometers with a cell sorting feature can also isolate or sort cells of interest into purified populations for further study [39]. 

### 3.4. Assessing T-Cell Function

Functional assays of T-cell function include measuring proliferation, cytokine production, and cytotoxicity [40]. A characteristic of the acquired immune response is the ability of lymphocytes specific to an antigen to proliferate and differentiate upon activation, which is often referred to as clonal selection [21]. Upon activation, helper T-cells produce and secrete cytokines that stimulate the activity of other immune cells, while cytotoxic T-cells kill the target cell when activated [24]. For activation, cytotoxic T-cells require the antigen to be combined with class I MHC, which is present on all nucleated cells [25]. An antigen activates the T-cell receptor (TCR) on helper T-cells only when it is processed into peptides and presented as a complex with a class II (MHC) protein, which is expressed by antigen presenting cells like B-cells, macrophages and dendritic cells [25]. 

T-cells can be characterized based on their functions, and this has lead to designations such as Th1/Th2, Th17, T-regulatory, *etc*. Classically, helper T-cells have been classified based on the cytokines that they produce into Th1 and Th2 cells. Th1 cells produce interleukin (IL)-2 and interferon (IFN)-γ, which are effective at stimulating the cell mediated immune response to fight off intracellular invaders while Th2 cells produce IL-4 and IL-10 which stimulate the humoral immune response which is more effective for fighting off intercellular pathogens [41]. The cytokines produced by Th1 and Th2 cells inhibit each other and ensure that the immune response is not excessive [42]. Therefore, an imbalance in the Th1 to Th2 cells could result in autoimmune diseases (overactive Th1) or allergies (overactive Th2) due to an uncontrolled immune response [43]. 

## 4. Experimental Design Considerations

### 4.1. Animal Model

The World Health Report in 2002 identified zinc deficiency as one of the major causes of disease, especially in developing countries [20]. Children are one of the segments of the population that are at particular risk [20]. However, studies investigating the impact of dietary zinc deficiency on the immune system have largely been done using the adult mouse as a model [44]. The mouse has been a convenient model because the necessary antibodies were readily available. However, mice are not amenable to studying the effects of dietary treatments during growth because they achieve adult size quickly and there is relatively little difference between body weight during infancy and adulthood [45]. Another limitation of their size is it can be difficult to remove enough tissue, including blood, from one animal to use for all the desired laboratory tests. 

The use of the growing rat model for studies of dietary zinc deficiency on T-cell development/function is now possible due to increased availability of the necessary antibodies. The use of the growing rat as a model in dietary zinc deficiency allows the researcher to explore the effects of this nutritional deficiency at an earlier stage of development. Rats can provide more tissue per animal, which allows for multiple tests on one animal, providing a more holistic picture of what is happening *in vivo*. It is of great interest to identify and examine recent thymic emigrants since it remains unclear whether the immunodeficiency of dietary zinc deficiency is due to a defect in T-cell maturation in the thymus or in the periphery. In humans and mice, recent thymic emigrant cells can be identified by T-cell receptor excision circle (TREC) content along with cell surface markers such as CD31 and CD45RA [46]. The rat offers the unique opportunity to explore the effect of dietary zinc deficiency on the newly produced T-cells using CD90 as a cell surface marker [30]. In the rat, immature peripheral T-cells can be further characterized as recent (0–1 day) and late (1–3 days) thymic emigrants based on their expression of CD90, CD45RC and RT6.1 [47].

### 4.2. Degree of Dietary Zinc Deficiency and Pair-Fed Controls

A potential confounding factor in studies examining the effects of dietary zinc deficiency on the immune system is that the consumption of zinc deficient diet results in significant reductions in feed intake which produces stunting malnutrition in growing animals [48] or wasting malnutrition in adult rodents [49]. Energy malnutrition has a negative impact on the ability of the body to fight off disease [50]; therefore, an additional control must be included in research studies to separate the effects of malnutrition from zinc deficiency. To control for the effects of malnutrition, researchers often individually match a “pair-fed” mouse (or rat) to a zinc deficient mouse and feed the pair-fed mouse (or rat) the control diet, but limited to the amount of feed as consumed by its zinc deficient paired mate on the previous day [51]. Given that the zinc requirement for rats is 12 mg Zn/kg diet [52], the reduced feed intake (approximately 50% for growing rats) of diet with adequate zinc (30 mg Zn/kg diet) based on the recommendations of the American Institute of Nutrition-93G diet [53] by the pair-fed group still results in sufficient zinc intake. Although the pair-fed approach and matching feed intake works well for the rat model, there have been reports that the feed intake of zinc-deficient mice is not different from control mice despite a difference in body weight [54]. In this situation, a pair-weighed group (diet restriction to match body weight) may be more appropriate to control for the lack of growth relative to baseline for zinc-deficient mice and the metabolic changes associated with the malnutrition [55]. 

The inclusion of a pair-fed (or diet- or energy-restricted) group is critical in studies of dietary zinc deficiency to isolate the effects of zinc deficiency from the accompanying malnutrition [51]. Another valuable control is a marginally zinc-deficient group, which receives a diet low in zinc, but not sufficient to induce anorexia and weight loss [56]. The marginally zinc-deficient group in combination with the pair-fed group can be used to separate the effects of low zinc status from malnutrition. Because severe zinc deficiency is rare in the human population, the marginal zinc deficient group represents the degree of zinc deficiency which is more prevalent.

### 4.3. Assessment of Primary and Secondary Lymphoid Organs

Due to the easy access and preparation of splenocytes from spleen and the focus on the response of immune cells in the periphery, much of the research on nutritional deficiencies and immune cells has focused on splenocytes. However, studies of dietary zinc deficiency on T-cell development and function should investigate both thymocytes and T-cells from secondary lymphoid organs (e.g., spleen, lymph nodes, gut associated lymphoid tissue) to determine whether any changes in T-cell subsets occurs during the maturation process in the thymus or once the cells have been released into the periphery. Immunological studies in rodent models should also investigate whether the effects in the cells isolated from lymphoid tissue are reflected in immune cells isolated from blood as this will aid in the translation to human studies where this is usually the only tissue that can be sampled. 

## 5. Dietary Zinc Deficiency and Cell-Mediated Immunity

The following sections will consider some of the consequences of dietary zinc deficiency on characteristics of the cell-mediated immune system and potential mechanisms.

### 5.1. Dietary Zinc Deficiency and Lymphopenia

Like humans, zinc deficient mice are less able to fight off infection compared to zinc adequate controls [57,58]. In one of the early reports of dietary zinc and immune function, Fraker and colleagues found that zinc deficient mice weighed 30% less than controls, and that their spleen and thymus weighed 50% and 70% less than controls, respectively [59]. Reduced lymphoid organ weight suggests reduced cell numbers which theoretically would result in fewer immune cells available to fight off infection. Subsequent studies have shown that zinc-deficient mice do indeed have fewer splenocytes [58] and thymocytes [60] which could contribute to the immunodeficiency. Both of these papers reported total splenocyte [58] or total thymocyte [60] numbers and did not take into account whether the differences were maintained when corrected for the respective reductions in spleen or thymus weight.

In the growing rat model, absolute thymus weight and spleen weight were substantially reduced in zinc deficient and pair-fed rats compared to *ad libitum* controls, but there was no thymic or splenic atrophy when lymphoid organ weight was expressed relative to body weight [61,62]. Similarly, absolute numbers of total T-cells (determined by flow cytometry and Flow Count™ fluorospheres) in thymus, spleen and blood were not different when corrected for lymphoid organ weight or µL blood, indicating a lack of lymphopenia relative to organ weight or blood volume in the growing rat model [62]. 

These differences in lymphopenia between the adult mouse and growing rat model may be due in part to different types of malnutrition. The zinc deficient adult mouse results in wasting malnutrition, while the zinc deficient growing rat results in stunting malnutrition. Wasting and stunting malnutrition have different effects on the immune system [63]. This should be taken into account when selecting an appropriate model for zinc deficiency and subsequently extending the findings to the population of interest. 

Both the young zinc deficient rat [62] and adult mouse [59] have the ability to recover lymphoid organ weight and cell numbers with dietary zinc repletion; however, future studies need to assess the functional ability of these cells including the resistance to infection. Nutritional repletion of zinc deficient and pair-fed (energy-restricted) rats with a zinc adequate control diet (30 mg Zn/g diet) resulted in faster recovery of spleen weight and splenic T-cell subsets (by 3 days) than thymus weight and thymic T-cell subsets (by 7 days except 23 days for thymus weight in zinc deficient rats); however, body weight was not recovered by 23 days [62]. Thus, there appears to be a priority for recovering lymphoid tissue before body weight allowing for enhanced production of T-cells for immune defense while nutritional recovery is in progress. The increase in cell numbers probably reflects replication of existing cells as T-cell maturation takes approximately 3 weeks in rodents [64]. Supplemental zinc in excess of the recommendations can result in reduced copper status [65], therefore, the finding that excessive amounts of dietary zinc are not necessary for recovery of lymphoid tissue is relevant to programs aimed at treating dietary zinc deficiency.

### 5.2. Dietary Zinc Deficiency and T-Cell Function

The reduced ability to resist infection in zinc deficiency could be due solely to lower lymphocyte numbers (when not corrected for body weight), or it could be due to a compromised functional ability of the existing cells. A study by Shi and colleagues [58] in mice has shown on a per cell basis lower proliferation rates and cytokine production by T-cells in response to antigen in zinc deficiency compared to a pair-fed group and *ad libitum* fed controls. Splenocytes isolated from zinc deficient growing rats produced less interleukin-2 (IL-2) in response to the mitogen Concanvalin A compared to pair-fed, marginally zinc deficient and *ad libitum* fed controls [66]. This provides evidence that not only are total lymphocyte numbers reduced, but their ability to respond to antigens is also compromised in dietary zinc deficiency. 

Some recent studies are beginning to elucidate the molecular basis for how zinc is involved in the response of T-cells to antigens. Based on *in vitro* addition of zinc to primary human T-cells and murine T-cell lines, IL-2 receptor stimulation results in increased intracellular concentrations of free zinc ions due to zinc release from lysosomes into the cytoplasm [67]. The increase in cytoplasmic zinc ions was necessary for IL-2 induced activation of ERK and cell proliferation [67]. Furthermore, it appears that after T-cell activation, zinc functions as an ionic signaling molecule [68]. The rise in intracellular zinc occurred within 1 min after T-cell receptor stimulation, was dependent on extracellular zinc concentrations, and required zinc influx via Zip6 [68]. T-cell proliferation in response to suboptimal stimuli could be promoted by increasing extracellular zinc in the media and thus zinc influx [68]. The next step will be studies to investigate how these zinc-dependent mechanisms apply to T-cell function in dietary zinc deficiency and supplementation *in vivo*. 

### 5.3. Dietary Zinc Deficiency and T-Cell Phenotypes

Alterations in T-cell phenotypes could explain the immune dysfunction present in zinc deficiency. One question has been whether zinc deficient rodents have an altered proportion of immune cell phenotypes which might explain the reduced functional abilities of the cells, or if the reduction in lymphocyte numbers is balanced across all cell types. Using CD3, CD4, and CD8 to identify T-cell subsets, no differences in T-cell phenotypes were found for thymocytes [66] or splenocytes [58] from zinc deficient mice. Likewise, there were no differences in the proportions of CD4^+^ or CD8^+^ T-cells in the thymus, spleen and blood of growing zinc deficient rats, except for a higher percentage of thymic CD8^+^ T-cells compared to control but not pair-fed rats [61]. Absolute numbers of some thymic and splenic T-cell sub-populations were reduced in zinc deficient rats compared to both pair-fed and *ad libitum*-fed control rats [splenic TCR^+^CD4^+^CD8^−^ (helper T-cells), splenic TCR^+^CD4^−^CD8^+^ (cytotoxic T-cells)] while other reductions occurred in both zinc deficient and pair-fed rats [thymic TCR^−^CD4^+^CD8^+^ and TCR^+^CD4^+^CD8^+^ (both pre T-cells)] indicating an effect of malnutrition. However, absolute numbers of total T-cells and T-cell sub-populations (determined by flow cytometry and Flow Count™ fluorospheres) were not different when corrected for lymphoid organ weight, indicating a lack of lymphopenia relative to organ weight in the growing rat model [62]. 

Overall, the research on T-cell phenotypes indicates that there are no major changes in the proportion of helper (CD4^+^) and cytotoxic (CD8^+^) T-cells in the growing rats or adult mice with zinc deficiency. Future studies need to investigate the expression of maturation and activation markers as demonstrated in the next section using CD90^+^ T-cells as the example.

### 5.4. Dietary Zinc Deficiency and Post Thymic T-Cell Maturation

In the rat, CD90 can be used to identify T-cells newly released from the thymus [30]. Interestingly, the growing zinc deficient rat has a lower proportion of splenic and blood CD90^+^ T-cells (new peripheral T-cells) when compared to *ad libitum* fed controls [61]. Furthermore, with dietary zinc repletion, the proportions of CD90^+^ T-cells in blood return to control levels within 7 days while proportions of CD90^+^ T-cells in spleen require 23 days of dietary zinc repletion to be restored. A subsequent study which used CD90, CD45RC and RT6.1 to explore peripheral T-cell maturation found that the first 2 stages of post thymic maturation do not appear to be affected, but it is the late thymic emigrants that are significantly lower with zinc deficiency [47]. The fact that the reductions in new T-cells in zinc deficient rats were observed in the periphery (cells from spleen and blood) but not thymus raises questions as to whether the “loss” of CD90^+^ cells in zinc deficient rats is due to faster maturation in the periphery, or greater susceptibility to apoptosis, or other unidentified factors. These results have significant implications as over time fewer new T-cells could adversely affect the T-cell repertoire, resulting in less diversity of T-cells and thus immune dysfunction. The concept of the T-cell repertoire has been of interest in diseases such as aplastic anemia, leukemia and graft-*versus*-host disease in humans [69] and more research is needed in the nutrition field to define the potential importance of the T-cell repertoire in understanding the consequences of immunodeficiency due to nutritional deficiencies such as zinc. 

### 5.5. Dietary Zinc Deficiency, Corticosterone and Apoptosis

A theoretical explanation for the lymphopenia that characterizes zinc deficiency discussed in the literature involves the hypothalamus-pituitary-adrenal stress axis [70]. High levels of steroids from the adrenals called glucocorticoids (cortisol in humans and corticosterone in rodents) can cause involution of the thymus by inducing apoptosis in thymocytes [71]. Apoptosis or programmed cell death is a form of cell suicide. Apoptosis occurs in an organized fashion whereby the nucleus and cytoplasm condense, chromatin is cleaved, and apoptotic bodies are removed by lysosomes without causing any significant inflammation [29]. Necrosis is another form of cell death that occurs when cells take up water until the plasma membrane bursts releasing the contents of the cell and usually results in initiation of an immune response [29]. Depasquale-Jardieu and Fraker [72] hypothesized that dietary zinc deficiency creates a stress in the animal, increasing the level of serum corticosteroids, and this explains the observed loss in numbers of functional thymocytes due to apoptosis. They reported that the zinc deficient mice had enlarged adrenal glands and higher plasma corticosterone concentrations (3.3 µM *vs.* 1.2 µM for control mice; [72]). Subsequently, it was reported that zinc deficient mice had an increased proportion of CD4^+^CD8^+^ thymocytes undergoing apoptosis compared to controls [60]. However, this study did not include a pair-fed group (malnourished mice also have elevated corticosterone levels compared to control [73]); therefore, it is not clear if the increased apoptosis is due to high corticosterone concentrations alone or if zinc deficiency has an additional effect. These researchers detected a loss in T-cell helper function in the zinc-deficient mice before any elevation in serum corticosterone suggesting that there are other factors besides the higher corticosterone concentrations associated with depletion of zinc that are compromising immune function [72]. Removal of adrenal glands did not offer significant protection against the immune dysfunction observed in zinc-deficient mice providing further evidence that corticosterone is not the only cause of the immunodeficiency in zinc-deficient mice [74]. 

Using the growing rat model, we have demonstrated that serum corticosterone concentrations were elevated 33–53 fold in both zinc deficient and pair-fed groups compared to *ad libitum* fed control rats [62]. Other components of the hypothalamus-pituitary-adrenal stress axis (serum ACTH, haptoglobin, and TNFα concentrations) were not affected by dietary zinc deficiency in the growing rat [62], thus, the cause of the malnutrition-induced elevation of serum corticosterone concentrations remains to be elucidated. For studies of dietary zinc deficiency, it is necessary to house the rats in stainless steel hanging cages to reduce zinc recycling. There is some concern that the hanging cages are stressful to the animals, however, the control rats had serum corticosterone concentrations that are well within the normal range (9.5 nmol/L). The rats were handled every day in order to limit the effect of handling on stress levels, and this was also reflected in the normal serum corticosterone concentrations in control rats. 

Despite the elevated serum corticosterone concentrations in both zinc deficient and pair-fed rats, adrenal gland weight corrected for body weight was elevated more in zinc deficient *vs.* pair-fed rats (65% *vs.* 34%) compared to *ad libitum* control rats [62]. The adrenal/body weight ratio of the pair-fed group recovered to control levels faster than the zinc deficient rats (3 days *vs.* 7 days, respectively). Although elevated serum corticosterone in both zinc deficient and pair-fed rats were associated with reduced numbers of some T-cell subsets per organ, there were no differences when T-cell subsets were expressed per gram of tissue [62]. In another study using the growing rat model which included a marginally zinc deficient (MZD) group with reduced zinc status, but no anorexia, MZD did not have elevated corticosterone concentrations or lymphoid organ atrophy, but they did have altered *ex vivo* mitogen-stimulated thymocyte cytokine production compared to controls [56]. These data in the growing rat model do not support the hypothesis that zinc deficiency *per se* is elevating corticosterone, promoting apoptosis of immune cells and resulting in low lymphoid organ cell numbers. Further studies directly assessing apoptosis and other mechanisms for apoptosis are still needed to evaluate the apoptosis theory in the context of zinc deficiency. 

It is also important to note that the thymic atrophy/lymphopenia associated with zinc deficiency may also be due in part to reduced thymulin activity. Thymulin is a hormone produced and/or stored by thymic epithelial cells [75] whose biological activity depends on zinc [76]. Thymulin stimulates IL-2 production and IL-2 receptor expression in T-cells (reviewed in [77]). Zinc deficiency leads to lower serum thymulin activity in mice and humans, which is corrected with zinc repletion [78,79]. 

### 5.6. Dietary Zinc Deficiency and P56^lck^

Another possible mechanism that could explain both the decreased number and decreased function of T-cells in zinc deficiency is an increased expression of the signaling protein p56^lck^ [6]. P56^lck^ is only expressed in lymphoid cells, and almost solely in T-cells [80]. P56^lck^ is part of the src-protein family, which is associated with the inside of the plasma membrane and CD4 or CD8 [81]. The cytoplasmic domains of CD4 and CD8 interact with the *N*-terminal of p56^lck^ through their cysteine residues as a “zinc clasp”-like structure [82,83]. The pairs of cysteine residues form a zinc-binding site, suggesting that zinc binding is necessary to stimulate p56^lck^ phosphorylation [84]. It is believed that once CD4 or CD8 stimulates p56^lck^, it phosphorylates the CD3 augmenting the TCR signals [85]. If the phosphorylation of this protein is inhibited, there is no activation of the T-cell [86]. 

Genetically altered mice missing p56^lck^ (lcknull) or those producing a catalytically inactive form of p56^lck^ (lckR257 transgenic mice) have thymic atrophy and a decreased number of thymocytes [87,88]. In both kinds of genetically altered mice there was a block in the maturation of thymocytes from double negative (the most immature) to double positive thymocytes suggesting that p56^lck^ plays a role in the maturation of thymocytes [87,88]. There was also a decrease in the number of peripheral T-cells, but the peripheral lymphoid tissues were not as severely affected as the thymus. Molina and colleagues [87] noted that B-cell function was not compromised in the lcknull mice. Levin and colleagues [88] found that thymocytes in lckR273 mice were stuck at a particular point in maturation and were unable to replicate past this point, suggesting that the decreased amount of thymocytes was due to less production and not more destruction. As well, p56^lck^ has also been shown to be involved in the signaling of apoptosis. Di Somma and colleagues [89] found that a Jurkat cell line defective for p56^lck^ was resistant to apoptosis, while another cell line with an active form of p56^lck^ that could not be turned off (constitutively expressed) enhanced the sensitivity of Jurkat cells to apoptosis induced by TCR crosslinking. Interestingly, the immunodeficiency in zinc deficiency has been proposed to be due in part to altered thymocyte development and/or increased susceptibility of immune cells to apoptosis, and this has led to the hypothesis that p56^lck ^may be a key player for explaining the molecular mechanism of immune dysfunction in dietary zinc deficiency [6].

Lepage *et al*. [55] were the first group to report elevated p56^lck^ protein levels in splenic T-cells from zinc deficient mice, and this was subsequently confirmed by Moore *et al*. [66] using a microarray approach. Thymocytes have 2-fold higher protein levels of p56^lck^ compared to splenocytes [61]. Both the zinc deficient adult mouse and the zinc deficient growing rat have elevated p56^lck^ protein levels in the thymus compared to *ad libitum* fed controls, but the zinc deficient growing rat did not have elevated splenic p56^lck^ protein levels as observed in the zinc deficient adult mouse [55,61,66]. This could be due to methodological differences. In the study with the zinc deficient rats, Western blotting of p56^lck^ used splenocytes [66] whereas the study with zinc deficient mice specifically used T-cells which were isolated by immunocolumns [55]. Perhaps the p56^lck^ protein levels are also elevated in the splenic T-cells of zinc deficient growing rats but differences were not detected due to the presence of other mononuclear cells in the spleen. 

Based on the available data, it appears that the elevated p56^lck^ levels in the thymus are not altering maturation of thymocytes from double negative (CD4^-^CD8^-^) to double positive (CD4^+^CD8^+^), as both the zinc deficient adult mouse and growing rat models do not show major alterations in their respective thymic T-cell subsets using CD4 and CD8 cell surface labeling [56,58,61,62,66]. Another consideration for future studies is the age-related changes in p56^lck^; protein levels of p56^lck^ decrease with age in thymic lymphocytes and increase with age in splenic lymphocytes from 3 to 9 week old rats [90]. A higher percentage of double negative (CD4^−^CD8^−^) and a lower percentage of DP (CD4^+^CD8^+^) cells in the thymus from 9 week old compared to 3 week old rats coincided with the decrease in thymic p56^lck^ levels. There is also some evidence that strong TCR signals amplified by p56^lck^ contribute to CD4 or CD8 lineage choice, and preferential differentiation of double positive cells to CD4 in mice and to CD8 in rats when cells are stimulated *in vitro* [91,92]. Interestingly, 9 week old rats had a higher proportion of thymic single positive CD8 cells compared to 3 week old rats [90], perhaps reflecting higher thymocyte p56^lck^protein levels in the younger rats and the time course for T-cell maturation (approximately 3 weeks upon entering the thymus [64].

Future studies should examine whether the function of p56^lck^ is altered by zinc deficiency. The function of p56^lck^ could be evaluated experimentally by measuring the level of phosphorylated p56^lck^ after stimulation of T-cells. The effects of elevated p56^lck^ on downstream functional outcomes such as proliferation or cytokine production could be evaluated by stimulating isolated T-cells with different mitogens (e.g., ConA stimulates via the TCR and phytohemagglutinin stimulates independent of TCR) to determine if the TCR signaling pathway (which involves p56^lck^) is altered by zinc deficiency. Although there are some assessments of proliferation and cytokine production in zinc deficiency, these have not been investigated concurrent with p56^lck^. The increased p56^lck^ levels in zinc deficiency could possibly be an attempt to compensate for decreased function of this protein, and decreased activity of p56^lck^ would explain the compromised functioning of T-cells in dietary zinc deficiency. However, if the elevated p56^lck^ levels do result in more activity of the protein, then this would potentially leave the T-cells more susceptible to apoptosis. Future studies need to determine whether there is an association between p56^lck^ and apoptosis in dietary zinc deficiency, and whether it is affected by stage of maturation, site of T-cells (thymic, splenic, *etc*.), age and/or species. 

## 6. Summary

There have been several advancements in the field since the seminal report characterizing effects of zinc deficiency on immune parameters in mice. The application of flow cytometry and access to antibodies has been important for characterizing cell numbers and phenotypes. The growing rat model of zinc deficiency (stunting malnutrition) has provided some new interpretations compared to the adult mouse model of zinc deficiency (wasting malnutrition). Notably, in the growing rat model of zinc deficiency, there is not the thymic or splenic atrophy relative to body weight or lymphopenia relative to organ weight or per µL blood. Through the use of both pair-fed and marginally zinc deficient controls, the previously held dogma that corticosterone concentrations were the cause of the impaired immune function associated with dietary zinc deficiency has been challenged. Both models do show signs of T-cell dysfunction; however, the mechanism of this functional loss should be explored further. Potential hypotheses include impaired p56^lck^ signaling, over active p56^lck^ signaling, and a decrease in the proportion of new T-cells leading to a more limited T-cell repertoire. Dietary zinc deficiency can have major effects on the immune system; however, the changes do not need to be permanent. Repletion with diet containing adequate zinc concentrations (not supplemental levels) leads to recovery of lymphoid tissue. Undoubtedly, further research is needed to identify the critical steps of immune function affected by zinc deficiency and to pinpoint the defect(s) due to zinc deficiency *per se*.
